# Artificial Intelligence (AI) in Home-Care and Community Nursing: A Scoping Review

**DOI:** 10.3390/healthcare14183034

**Published:** 2026-09-16

**Authors:** Haiying Wang, Grace Y. Wang, Tao Wang, Lori Delaney, Jonathan Bayuo, Mengyuan Li, Jing-Yu (Benjamin) Tan

**Affiliations:** 1 School of Nursing and Midwifery, University of Southern Queensland, Ipswich, QLD 4305, Australia; alison.wang@ecu.edu.au (T.W.); lori.delaney@unisq.edu.au (L.D.); jonathan.bayuo@unisq.edu.au (J.B.); mengyuan.li@unisq.edu.au (M.L.); benjamin.tan@nd.edu.au (J.-Y.T.); 2 Institute for Health, University of Southern Queensland, Springfield, QLD 4300, Australia; grace.wang@unisq.edu.au; 3 School of Health, Psychological and Medical Sciences, University of Southern Queensland, Ipswich, QLD 4305, Australia; 4 School of Nursing and Midwifery, Edith Cowan University, Joondalup, WA 6027, Australia; 5 School of Nursing and Midwifery, The University of Notre Dame Australia, Fremantle, WA 6160, Australia

**Keywords:** artificial intelligence, home-care nursing, community nursing, remote monitoring, implementation challenges, scoping review

## Abstract

**Background/Objectives**: Artificial intelligence (AI) is increasingly being applied across healthcare, but empirical evidence on its use in home-care and community nursing remains fragmented across technologies, clinical contexts, nursing roles, and stages of implementation. This scoping review aimed to map current AI applications in home-care and community nursing and synthesize reported implementation experiences, benefits, challenges, and future considerations. **Methods**: Guided by Joanna Briggs Institute methodology and reported according to PRISMA-ScR, eight databases and grey literature via ProQuest were searched for English-language empirical studies published from 1 January 2020 to 14 May 2026. Retrieved records were screened independently by two reviewers, with discrepancies resolved through discussion or consultation with a third reviewer. Data were charted using a pre-designed and pilot-tested extraction form and synthesized descriptively and thematically. **Results**: The searches identified 3254 records; after duplicate removal, 1085 records were screened, 38 full-text reports were assessed for eligibility, and 16 studies were included. AI applications were grouped into diagnostic and clinical assessment support, remote monitoring and risk prediction, and nursing workflow optimization and care coordination. Most evidence remained early-stage, heterogeneous, and context-specific, with many studies focused on development or validation rather than routine real-world implementation. Reported benefits included support for clinical assessment, proactive monitoring, chronic disease follow-up, and perceived workload reduction. Key challenges included limited external validation, small or context-specific datasets, interoperability barriers, privacy and accountability concerns, algorithmic bias, digital literacy needs, and potential risks to relational and person-centered nursing care. **Conclusions**: AI may support home-care and community nursing by strengthening assessment, monitoring, and coordination; however, current evidence should be interpreted as emerging rather than definitive because most included studies were early-stage, heterogeneous, and context-specific; therefore, the findings do not support broad claims of routine clinical effectiveness. Future research should prioritize equity-focused and large-scale real-world validation, implementation frameworks, interoperability, transparent governance, ethical safeguards, and nursing workforce preparation before widespread integration into community practice.

## 1. Introduction

In the context of global population aging and the rising prevalence of chronic health conditions, healthcare systems are undergoing substantial transformation [1]. Driven by these demographic and epidemiological trends, home-care and community nursing have become increasingly central to contemporary health systems [2]. The shift in care provision from hospitals to long-term management in community and home settings has further increased the demand for community and home nursing services [3]. Compared with hospital-based care, home and community nursing place greater emphasis on continuity of care, preventive interventions, chronic disease management, and patient-centered support delivered outside acute care facilities [4]. Within this context, nurses play a critical role in monitoring health status, coordinating services, and supporting the long-term management of complex conditions [4]. However, community and home care nursing is often delivered in diverse, dynamic, and resource-constrained environments. Unlike hospital settings, community nurses frequently work independently with limited immediate access to specialist support, while patient homes vary substantially in physical and social conditions. In addition, digital literacy, infrastructure, and internet connectivity differ considerably across communities [5,6]. These challenges may hinder care quality, efficiency, and patient safety, highlighting the need for innovative approaches to support care delivery in community and home settings [7,8].

To address these challenges, digital health technologies are evolving rapidly, with AI increasingly being integrated into healthcare over the past decade [9]. In home-care and community nursing, AI is relevant because it may help respond to several challenges outlined above, including limited specialist support, geographic dispersion, increasing chronic disease burden, and variable service capacity. AI-enabled tools, such as remote monitoring systems, wearable sensors, predictive analytics, conversational agents, robotics, smart-home technologies, and natural language processing applications, can support early risk detection, clinical decision support, patient education, documentation, communication, and care coordination [6,8,10]. These functions may enhance nurses’ capacity to identify subtle deterioration, tailor self-management support, and deliver more proactive and preventive care outside acute care facilities [7,8]. However, AI implementation in home-care and community nursing is not simply a technical issue. Its value depends on whether technologies are clinically meaningful, acceptable to nurses and patients, ethically governed, integrated with existing workflows, and equitable across diverse populations. Challenges related to data privacy, algorithmic bias, transparency, accountability, clinician acceptance, digital literacy, infrastructure, and preservation of relational nursing care remain important considerations for safe and effective implementation [11,12].

Understanding how AI technologies are currently used, implemented, and evaluated in home-care and community nursing practice is therefore essential [5,11]. The rapid pace of AI development makes it difficult for nurses, service managers, policymakers, and technology developers to maintain an integrated understanding of the current evidence landscape. Existing research is heterogeneous and dispersed across nursing, gerontology, health informatics, rehabilitation, public health, and computer science [13,14,15,16,17]. It includes qualitative studies exploring nurses’ and patients’ experiences, feasibility and prototype evaluations, prediction-model development studies, service evaluations of remote monitoring programs, and mixed-methods implementation research [18,19,20,21,22]. This diversity makes it difficult to determine which AI technologies are being applied, whether they are at development, validation, or implementation stages, and how they affect nursing workflows, professional judgement, patient safety, equity, and person-centred care.

Several recent reviews have examined AI technologies in community- and home-based care, but important gaps remain. Some reviews have focused on specific populations, clinical areas, or technologies, such as wound care, smart-home systems, older adult care, rehabilitation, or broader AI adoption in healthcare [23,24,25,26,27]. Rahmadhani et al. and Wibisono et al. specifically reviewed AI in community and home nursing care [25,26]. These reviews provide useful background; however, they offer limited differentiation between nursing-specific and broader healthcare applications and provide less detailed synthesis of implementation processes, nursing roles, technology maturity, socio-technical barriers, ethical implications, and future implementation requirements. In addition, previous reviews draw partly on secondary evidence or broad descriptions of AI technologies, whereas the present review focuses on empirical primary studies directly relevant to home-care and community nursing. A scoping review is therefore appropriate because the field is emerging, heterogeneous, and not yet sufficiently mature for effectiveness-focused synthesis alone. This review maps the extent and nature of available empirical evidence, clarifies how AI is being used or tested in nursing-related home and community contexts, and identifies gaps requiring further research, implementation planning, and policy attention.

Accordingly, the primary objective of this scoping review was to systematically map empirical evidence on AI applications in home-care and community nursing. The secondary objectives were to describe how these technologies have been implemented or tested, synthesize reported benefits and implementation experiences, identify barriers, facilitators, challenges, and limitations, and clarify implications for future development, implementation, and evaluation. Specifically, the review addressed the following questions:

1. What AI technologies are currently being used or tested in home-care and community nursing?
2. How are these technologies implemented, and what benefits or intended functions have been reported?
3. What barriers, facilitators, challenges, and limitations have been reported in implementing AI within home-care and community nursing?
4. What implications and key considerations can be synthesized from the current evidence to inform future development, implementation, and evaluation of AI in home-care and community nursing?

## 2. Materials and Methods

Given the emerging, interdisciplinary, and heterogeneous nature of the evidence, a scoping review was considered the most appropriate approach to map existing research on AI in home-care and community nursing. Scoping reviews are particularly useful when the purpose is to examine the extent, range, and characteristics of research activity rather than to determine intervention effectiveness or conduct meta-analysis [28]. This approach was suitable because the included evidence was expected to vary across study designs, AI technologies, care settings, populations, and stages of implementation. The review was conducted in accordance with JBI methodological guidance for scoping reviews and reported using the PRISMA-ScR checklist [29,30]. The protocol was retrospectively registered with the Open Science Framework (OSF) and is available at: https://osf.io/u8gvn/overview, accessed on 11 July 2026 Retrospective registration is acknowledged as a transparency limitation because it does not provide the same assurance as prospective registration; however, the review was guided by predefined Population, Concept, and Context (PCC) eligibility criteria, a structured search strategy, independent screening procedures, and a pilot-tested data-charting form.

### 2.1. Eligibility Criteria

PCC eligibility criteria were used in this review [30].

Population (P): Primary empirical studies were eligible if they focused on registered or practicing nurses delivering home-care or community-based services; patients, older adults, or family caregivers receiving AI-supported nursing care; or health professionals, managers, technology providers, or organizational stakeholders involved in implementing AI-supported home-care or community nursing services. Organizational or multidisciplinary participants were included when their role was directly related to AI-enabled nursing care delivery, service coordination, documentation, or implementation in home-care or community contexts. Studies were included only when nursing practice, nursing documentation, nurse-led or nurse-involved service delivery, or community/home-care nursing workflows were central rather than peripheral to the AI application.

Concept (C): Studies were eligible if they described, developed, validated, evaluated, or implemented technologies explicitly reported as AI or involving AI-related methods. These included machine learning prediction tools, deep learning or image-recognition systems, wearable devices and remote monitoring systems with AI-enabled analytics, conversational agents, chatbots or virtual nurses, robotics used in nursing care, smart-home systems with AI features, natural language processing applications for nursing documentation, and AI-driven decision-support systems. Technologies were not classified as AI solely because they were digital; the included studies needed to report AI-related functions such as prediction, classification, pattern recognition, natural language processing, adaptive decision support, or automated learning from data.

Context (C): Studies were included if they were conducted in home-care or community nursing contexts, including home visits, home health or home nursing services, community-based chronic disease care, in-home aged care, community health service centers, primary care or community-based follow-up services where community or home-care nurses were involved. The review focused on care delivered outside acute hospital settings and included diverse models of care when the AI application was relevant to nursing assessment, monitoring, coordination, education, documentation, or long-term management in home or community environments. Quantitative, qualitative, and mixed-methods empirical studies were eligible. Grey literature, such as dissertations and theses, and conference papers reporting empirical data were also considered. Studies were limited to English-language publications from 2020 onwards to capture contemporary AI applications in this rapidly evolving field.

Studies were excluded if they focused only on student nurses; did not involve nurses, nursing documentation, or nursing-related workflows; were conducted solely in hospital or acute-care settings without a home-care or community nursing component; described non-AI digital health tools; were purely technical or computer-science studies without clinical or nursing relevance; or reported algorithm development without any connection to home-care or community nursing practice. Reviews, editorials, commentaries, letters, protocols, and retracted publications were excluded from the evidence synthesis, although relevant reviews were used to contextualize the background and discussion.

### 2.2. Search Strategy and Study Selection

A comprehensive search was conducted on 14 May 2026 in PubMed, CINAHL, Web of Science, Embase, Scopus, Ovid, and PsycINFO. Grey literature, including dissertations and theses, was searched via ProQuest One Academic. Search terms combined controlled vocabulary, where available, with keywords and free-text terms related to AI, nursing, home care, and community care. Examples included “artificial intelligence”, “machine learning”, “deep learning”, “chatbot*”, “natural language processing”, “nurs*”, “home care”, “home health”, “community nursing”, and “community care”. Search strategies were adapted for each database. The full database search strategies are provided in Supplementary File S1.

**Figure 1:**
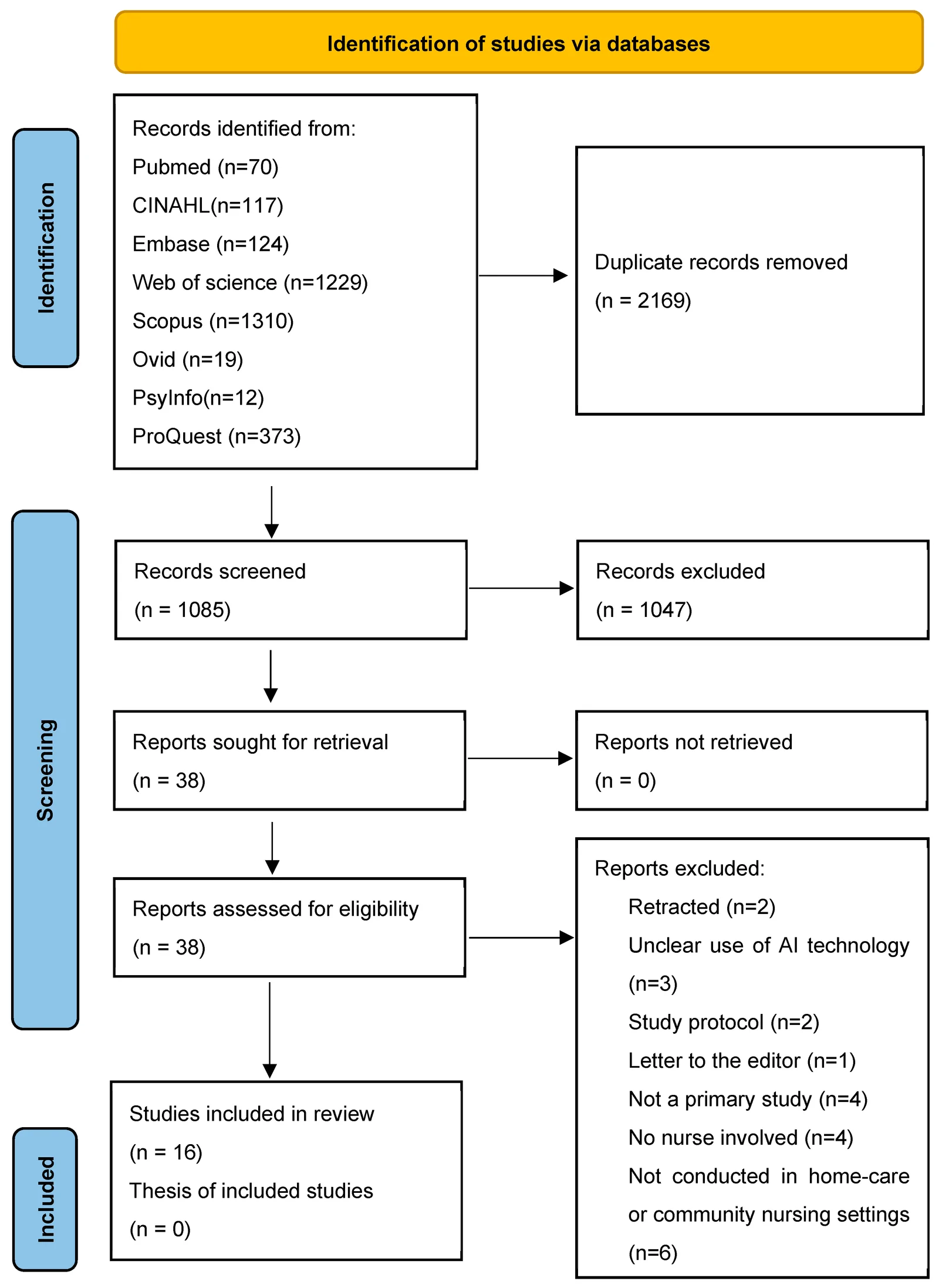
PRISMA Flow Chart. This work is licensed under CC BY 4.0. To view a copy of this license, visit https://creativecommons.org/licenses/by/4.0/, accessed on 13 July 2026.

All retrieved records were imported into EndNote 21.3 (Clarivate, Philadelphia, PA, USA), and duplicates were removed before screening. Two reviewers (H.W. and M.L.) independently screened titles and abstracts against the eligibility criteria. The same reviewers then independently assessed the full texts of potentially eligible studies. Disagreements at either stage were resolved through discussion; where consensus could not be reached, a third reviewer (T.W.) was consulted. A formal inter-rater reliability statistic was not calculated because the purpose of screening in this scoping review was to ensure comprehensive mapping of the evidence rather than to estimate agreement as an outcome. However, independent screening, reviewer discussion, and third-reviewer adjudication were used to enhance consistency and transparency. The study selection process is presented in the PRISMA flow diagram (Figure 1).

### 2.3. Data Extraction

Data were extracted from included studies using a pre-designed charting form developed to capture information aligned with the review objectives. Extracted data included author, year, country, study design, aims, setting, sample size, participant or stakeholder characteristics, nursing or caregiver roles, type of AI technology, purpose and function, stage of technology maturity or implementation, key findings, reported challenges or limitations, and implications for nursing practice (Table 1). The charting form was pilot-tested on a subset of studies and iteratively refined to improve clarity, consistency, and comprehensiveness. The finalized data extraction table is included in the manuscript and may also be submitted as Supplementary Materials if required by the journal.

### 2.4. Data Analysis and Synthesis

Data were analyzed using a structured descriptive and thematic synthesis approach consistent with JBI guidance for scoping reviews [30]. Extracted data were first charted in a standardized matrix to summarize study characteristics, countries, care settings, participant groups, AI technologies, study designs, implementation approaches, and reported outcomes. Because the included evidence was heterogeneous and comprised qualitative interviews, surveys, observational studies, feasibility and prototype evaluations, model-development or validation studies, and experimental designs, findings were synthesized narratively rather than statistically. No meta-analysis was planned or conducted.

A descriptive summary was developed to map the distribution of AI applications across home-care and community nursing contexts, including technology type, country, clinical focus, nursing involvement, and stage of maturity. Studies were also considered according to whether the AI application was primarily at the development, validation, or real-world implementation/clinical evaluation stage, so that simulation-based or prototype studies were not interpreted as equivalent to technologies implemented in routine care. An inductive thematic synthesis was then conducted to identify patterns related to current AI applications, implementation experiences, reported benefits, barriers, facilitators, challenges, and implications for nursing practice. The whole review team contributed to the interpretation of the charted data. Preliminary codes and categories were discussed, regrouped where concepts overlapped, and refined through iterative comparison across studies until consensus was achieved. Disagreements were resolved through team discussion. The synthesis focused on characterizing the breadth of existing evidence and identifying practical, ethical, implementation, and research implications for AI in home-care and community nursing.

## 3. Results

### 3.1. Search Results

The database and grey-literature searches identified 3254 records published between 1 January 2020 and 14 May 2026. After 2169 duplicate records were removed, 1085 records were screened by title and abstract, and 1047 records were excluded. Thirty-eight full-text reports were assessed for eligibility. Twenty-two reports were excluded because they were retracted (n = 2), did not clearly involve AI technology (n = 3), were study protocols (n = 2), were letters to the editor (n = 1), were not primary studies (n = 4), did not involve nurses or nursing-related care (n = 4), or were not conducted in home-care or community nursing settings (n = 6). Sixteen studies met the eligibility criteria and were included in this scoping review. The study selection process is presented in Figure 1.

### 3.2. Study Characteristics and Populations

The 16 included studies represented an international evidence base, with studies conducted in China, South Korea, the United Kingdom, Sweden, the Netherlands, the United States, Brazil, Canada, Lesotho, and Türkiye. This distribution indicates growing global interest in AI-enabled home-care and community nursing, although the evidence was geographically uneven and concentrated in a relatively small number of countries. Several studies originated from Asian contexts, particularly China and South Korea, while fewer studies were conducted in low- and middle-income or rural and remote settings. This geographical pattern suggests that future research should examine the transferability of AI applications across health systems, community-care models, and resource settings.

The 16 included studies [23,31,32,33,34,35,36,37,38,39,40,41,42,43,44,45] were heterogeneous in design and included experimental or quasi-experimental studies [31,32], cross-sectional or survey-based studies [33,36,43], retrospective or observational studies [34,39,41], qualitative or exploratory studies [23,42], and AI model development, validation, or evaluation studies [35,37,38,40,44,45]. All studies were situated in home-care or community-based care contexts, including home health agencies [41], municipal home healthcare [23], community health service centers [35], community-based follow-up services [32,42], and remote or home-based monitoring platforms [38,40]. Populations included older adults, people living with chronic or complex conditions, patients receiving post-discharge or follow-up care, nurses, and community or visiting healthcare professionals. Nursing involvement was central across the included studies, either through direct delivery of home- or community-based interventions [31,32], nurse participation in evaluating technology acceptance or usability [33,36,43], nursing workflow or service coordination [23,42], or use of nursing notes and nursing documentation for AI model development and prediction [37,41,44]. Sample sizes varied substantially, from small clinical or feasibility studies to large datasets containing thousands of images or records, reinforcing the heterogeneity of the evidence base. The characteristics of the included studies are summarized in Table 1.

When considered by stage of technology maturity, the included studies ranged from early development to validation and limited real-world implementation. Development-stage studies included AI models or systems tested in simulated, prototype, or preliminary contexts, such as AI-assisted incentive spirometry or predictive modelling approaches [34,38]. Validation-stage studies assessed model performance, transferability, or classification accuracy using clinical, documentation, image, or cohort datasets [35,37,39,40,44]. A smaller number of studies reported implementation or clinical evaluation in community or home-care contexts, including AI-supported continuous care platforms, AI-assisted focused echocardiography in community screening, and digital platforms supporting visiting health professionals [32,42,45]. This maturity classification is important because development and validation studies provide evidence of technical feasibility, whereas implementation studies provide more direct insight into clinical integration, nursing workflow, acceptability, and potential patient or service outcomes.

### 3.3. Applications, Experiences, Challenges, and Limitations of AI in Home-Care and Community Nursing

#### 3.3.1. Current Applications of AI Technologies in Home-Care and Community Nursing

Across the included studies, AI technologies were primarily used to support clinical assessment, remote monitoring and risk prediction, and nursing workflow or care coordination. These domains should be interpreted as overlapping rather than mutually exclusive. Some technologies contributed to more than one function; however, studies were grouped according to their dominant purpose, reported aims, and primary nursing relevance.

(1) Diagnostic and Patient Assessment Support

Six studies supported diagnosis, classification, or patient assessment through image analysis, pattern recognition, predictive modelling, or AI-assisted interpretation [31,34,39,40,44,45]. Examples included AI-supported wound assessment using patient-submitted photographs [40], AI-assisted focused echocardiography for detecting left ventricular hypertrophy in a community-based cardiovascular screening context [45], and algorithmic screening for depression or anxiety using cognitive performance data collected remotely [39]. Although some diagnostic technologies were used in community or remote settings, they were categorized in this domain when their primary purpose was assessment or diagnostic classification rather than continuous monitoring over time. For example, the study by Firima et al. was classified as diagnostic and assessment support because its main focus was AI-supported echocardiographic identification of left ventricular hypertrophy, enabling nurses or trained community personnel to obtain interpretable images that could support cardiovascular assessment [45].

(2) Remote Monitoring and Risk Prediction

Six studies used AI to support remote monitoring, risk prediction, or follow-up in people living with chronic or complex conditions [32,33,38,39,41,44]. AI-assisted respiratory devices classified breathing performance to support home-based rehabilitation [38], while AI-enabled continuous care platforms supported post-discharge self-management and follow-up for stroke survivors [32]. Natural language processing and machine learning approaches were also applied to home health nursing notes to identify risk factors, poor self-management, or diabetes risk, demonstrating how routine nursing documentation may be used to support anticipatory community care [41,44]. Home-care nurses also expressed interest in a broader ecosystem of AI-enabled sensors, fall detection systems, medication support tools, and AI-IoT technologies that could enhance automated surveillance and timely response in home environments [33].

(3) Nursing Workflow, Care Coordination, and Operational Efficiency

Three studies focused primarily on nursing workflow, care coordination, or operational efficiency [23,42,43]. AI-enabled or digitally supported platforms were used to streamline referral pathways, scheduling, navigation, multidisciplinary communication, and service coordination for visiting health professionals [42]. Machine learning models using nursing notes also demonstrated potential to support nurses’ decision-making by identifying patients at higher risk and enabling more timely and individualized interventions [44]. However, the workflow benefits were not automatic. Nurses reported that technologies were more acceptable when they reduced duplication, improved information sharing, and aligned with existing care processes, whereas poorly integrated systems risked increasing administrative burden [23,43].

#### 3.3.2. Implementation Experiences

Implementation experiences were reported in a smaller subset of studies, and findings should therefore be interpreted cautiously. Five studies reported patient- or service-related outcomes associated with AI-supported technologies in home- or community-care contexts [31,32,38,39,45]. AI-supported continuous nursing was associated with improved physical independence and depression-related outcomes among stroke survivors receiving ongoing follow-up care [32], while AI-augmented nursing approaches were reported to support quality-of-life-related outcomes in people with diabetic nephropathy [31]. AI-assisted focused echocardiography suggested that community nurses or trained non-specialist staff could acquire interpretable images after brief hands-on training, potentially expanding access to cardiovascular assessment in community settings [45]. However, these findings do not establish definitive effectiveness because studies varied in design, sample size, context, intervention intensity, and outcome measurement. Current evidence is better understood as indicating feasibility, acceptability, and potential clinical value rather than conclusive evidence of sustained patient benefit.

Nurses’ perceptions of AI were mixed. Several studies suggested that nurses viewed AI as potentially useful for workload reduction, monitoring-intensive tasks, risk prediction, documentation support, and timely follow-up [33,36,43]. At the same time, concerns were raised about professional accountability, data security, trust, usability, and whether AI-generated recommendations could conflict with nurses’ clinical judgement [33,43]. These findings indicate that implementation success depends not only on technical performance but also on nurses’ confidence in the system, clarity of governance, integration into workflow, and preservation of professional judgement.

#### 3.3.3. Challenges and Limitations

Despite promising benefits, implementation challenges reflected a combination of technical constraints, nursing practice tensions, and regulatory and user-level barriers.

(1) Technical and Data-Related Challenges

Technical and data-related challenges were reported across several studies. Prediction models often relied on small datasets, simulated data, single-context samples, or early-stage prototypes, limiting generalizability to routine clinical practice [31,34,38,39]. Diagnostic performance was also sensitive to real-world conditions such as image quality, lighting, patient technique, device usability, and the variability of home environments [38,40,45]. Algorithm transparency, explainability, and fairness were additional concerns. For example, differential performance across skin tones in wound analysis raises equity and patient-safety concerns because algorithmic bias may contribute to delayed diagnosis, inappropriate escalation, or unequal access to accurate assessment [40]. These findings reinforce the need for external validation, representative datasets, bias assessment, explainable outputs, and user-centered design before AI tools are adopted widely in home-care and community nursing.

(2) Nursing Practice and Workload Barriers

Nursing practice and workload barriers reflected tensions between technological efficiency and the relational, embodied, and situational nature of nursing care. Although AI may reduce workload in monitoring-intensive tasks, nurses reported that some digital systems increased administrative burden when they were not well integrated into existing workflows [23]. Nurses also expressed concern that over-reliance on AI could weaken the human elements of nursing practice, including observation, touch, smell, communication, contextual judgement, improvisation in unpredictable home environments, and person-centered interaction [23]. Mandatory or poorly designed systems reduced satisfaction and intention to use technology when nurses perceived them as administrative rather than clinically meaningful [33]. These findings highlight the need for AI tools that augment rather than replace nursing judgement and that are co-designed with nurses, patients, and caregivers.

(3) Regulatory, Digital Literacy, and Integration Barriers

Concerns about data privacy, legal accountability, system security, digital literacy, and interoperability remained significant barriers to wider adoption [23,43]. In one quantitative study, perceived security protection was among the strongest predictors of nurses’ use of mobile home nursing care applications, ranking second in importance after outcome expectations, while ease of use also ranked highly [43]. This indicates that security, trust, and usability are core implementation determinants rather than peripheral concerns. Interoperability limitations and restrictive information-sharing policies also hindered efficient workflow, communication, and continuity of care among healthcare professionals [23]. These barriers should be understood as features of a complex socio-technical ecosystem rather than isolated technical problems. Without attention to governance, infrastructure, training, workflow integration, and cross-system communication, AI-enabled systems may increase workload or fragment care rather than support sustainable integration into community practice.

## 4. Discussion

This scoping review mapped empirical evidence on AI applications in home-care and community nursing and identified three broad areas of use: diagnostic and clinical assessment support, remote monitoring and risk prediction, and workflow optimization or care coordination. The findings suggest that AI may contribute to more proactive, data-informed, and preventive models of community nursing by supporting earlier identification of risk, chronic disease follow-up, documentation, and coordination across dispersed care environments. However, the evidence remains small, heterogeneous, and unevenly mature. Many studies focused on prototype development, model validation, or feasibility rather than sustained implementation in routine community nursing practice. Therefore, the findings should be interpreted as evidence of emerging opportunity and implementation need rather than definitive evidence of clinical effectiveness.

The review extends previous reviews by focusing specifically on empirical primary studies in which nursing practice, nursing documentation, or nurse-involved home and community care were central to the AI application. Rahmadhani et al. and Wibisono et al. provided useful overviews of AI in community and home nursing care [25,26], but their syntheses paid less attention to technology maturity, implementation context, nursing role boundaries, and socio-technical barriers. By distinguishing development and validation studies from real-world implementation studies, the present review shows that much of the literature remains at an early stage. This distinction is important because technical performance in a controlled or simulated environment cannot be assumed to translate into safe, acceptable, equitable, or sustainable use in complex home-care settings. Community nursing practice occurs in variable home environments, often with limited infrastructure, reduced real-time clinical support, and greater reliance on patients, caregivers, and nurses’ independent judgement. These contextual features mean that AI tools require evaluation not only for accuracy but also for usability, workflow fit, governance, patient safety, and impact on nursing relationships.

The findings also highlight the need to understand AI implementation as a socio-technical process. Implementation frameworks, such as the Consolidated Framework for Implementation Research (CFIR) and RE-AIM, provide useful lenses for translating AI from development to community practice [46,47]. Recent implementation research on healthcare AI has shown that routine adoption is influenced by factors across CFIR domains, including characteristics of the innovation, stakeholder engagement, data access, technical integration, resourcing, and the implementation process [48]. From a CFIR perspective, successful implementation will depend on the characteristics of the AI intervention, the inner setting of community nursing services, the outer policy and funding environment, individual nurses’ knowledge and confidence, and the implementation process itself [46,47,48]. From a RE-AIM perspective, future studies should report not only model performance but also reach across patient groups, effectiveness in real-world care, adoption by nurses and organizations, implementation fidelity and workflow burden, and maintenance over time [47]. Applying these frameworks would help move the evidence base beyond descriptions of technological promise toward more actionable guidance for implementation, scalability, and sustainability.

### 4.1. Implications for Practice and Implementation

For nursing practice, AI should be positioned as an adjunct to, rather than a replacement for, professional judgement. Recent nursing scholarship emphasizes that AI may support decision-making, diagnostic accuracy, and workflow efficiency, but safe integration requires transparency, usability, clinical alignment, and attention to nurses’ interpretive and ethical responsibilities [46,49,50,51,52,53,54,55,56,57,58,59]. Home-care and community nurses rely on communication, observation, clinical reasoning, contextual knowledge, and embodied assessment in environments that are less controlled than hospitals. AI-enabled monitoring, prediction, and assessment tools may support earlier escalation and more individualized follow-up, but they cannot replace the interpretive and relational work that is central to nursing. Implementation should therefore prioritize co-design with nurses, patients, caregivers, and service managers so that AI tools address clinically meaningful problems, reduce duplication, and complement existing care pathways. Training should include not only technical use but also interpretation of AI outputs, uncertainty, escalation procedures, documentation, ethical decision-making, and communication with patients and families [46,49,50,51,52,53,54,55,56,57,60].

### 4.2. Implications for AI Design in Community Settings

For technology design, transparency, explainability, usability, and interoperability are essential. Reviews of healthcare AI implementation and governance consistently identify fragmented data infrastructure, limited interoperability, lack of explainability, privacy and cybersecurity risks, unclear accountability, poor workflow integration, and insufficient external validation as major barriers to safe clinical adoption [48,56,61,62,63]. Nurses and patients are unlikely to trust AI tools if recommendations are opaque, difficult to interpret, or poorly aligned with clinical workflows. Explainable outputs, clear thresholds for action, integration with existing documentation systems, and mechanisms for nurses to override or question AI-generated recommendations are needed to preserve professional accountability. Interoperability is particularly important in home-care and community nursing because fragmented systems may increase documentation burden, disrupt communication, and undermine continuity of care. AI-enabled tools should therefore be embedded within broader digital health infrastructure rather than introduced as isolated applications [62,63].

Ethical implications require more than general statements about privacy and security. AI in home-care and community nursing often operates in private homes, where monitoring technologies may affect autonomy, dignity, consent, and perceptions of surveillance. Recent reviews of AI ethics in healthcare and nursing identify patient autonomy, privacy, accountability, equity, algorithmic bias, and nurse–patient relationships as key ethical domains requiring proactive governance [60,64,65,66]. Algorithmic bias is also a patient-safety issue. For example, reduced performance across skin tones in wound assessment may contribute to delayed recognition, inappropriate escalation, or inequitable care. Reviews of bias in healthcare AI emphasize that bias can arise across the AI lifecycle and may exacerbate existing health disparities if not systematically assessed, mitigated, and monitored after deployment [67,68]. From a nursing ethics perspective, such risks are directly relevant to non-maleficence, justice, and advocacy. Nurses need governance structures that clarify responsibility when AI outputs are inaccurate, incomplete, or inconsistent with clinical judgement. Ethical implementation should include representative datasets, bias testing, informed consent processes, privacy protection, cybersecurity safeguards, human oversight, and clear accountability pathways [60,64,65,67,68].

### 4.3. Research Gaps and Future Directions

Future research should move beyond early feasibility and model-performance studies toward rigorous, implementation-focused evaluation in real-world home-care and community nursing settings. Large-scale prospective studies, pragmatic trials, hybrid effectiveness–implementation designs, and longitudinal mixed-methods evaluations are needed to examine clinical impact, workflow effects, cost, safety, acceptability, equity, and sustainability [46,47,48]. Studies should report implementation outcomes such as adoption, acceptability, appropriateness, feasibility, fidelity, penetration, and maintenance, alongside patient, caregiver, nurse, and service-level outcomes. Where AI tools are designed for prediction or classification, future studies should include external validation across diverse datasets, transparent reporting of algorithm performance, calibration, subgroup performance, and bias assessment [63,67,68].

Equity should be treated as a core evaluation outcome rather than an optional consideration. Future studies should assess whether AI tools perform consistently across age, gender, ethnicity, skin tone, language, geography, socioeconomic status, disability, health literacy, digital literacy, and comorbidity profiles. This is particularly important in community nursing because patients receiving home-care services may be older, socially isolated, living with multimorbidity, or dependent on caregivers for technology use. Recent healthcare AI literature emphasizes that responsible AI adoption requires diverse and representative datasets, equity-oriented model development, bias mitigation strategies, and post-deployment surveillance to prevent the amplification of existing inequities [67,68]. Research should also examine how AI affects access to care in rural, remote, low-resource, and culturally diverse settings.

Further qualitative and participatory research is needed to understand how nurses, patients, caregivers, managers, and technology developers experience AI in home and community care. Such studies should explore trust, professional identity, perceived loss of human touch, patient dignity, caregiver burden, communication, and the practical work required to integrate AI into everyday care. Co-design approaches may help ensure that future AI systems are clinically meaningful, culturally appropriate, accessible, and aligned with nursing values. This is consistent with nursing-focused AI literature emphasizing that nurses should be actively involved in AI design and implementation so that technologies support, rather than undermine, clinical judgement, professional identity, and relational care [51,53,55,56,58,60].

Implementation-science-informed research is also required. Future studies should explicitly apply frameworks such as CFIR and RE-AIM to examine contextual readiness, organizational support, workflow integration, training needs, implementation strategies, reach, adoption, fidelity, and maintenance [58,64,65]. These frameworks can help researchers and service managers determine why an AI intervention succeeds or fails, for whom it works, under what conditions, and what resources are required for sustainable scale-up.

Finally, future research should include explicit governance and policy evaluation. Studies should examine practical models for data sharing, cybersecurity, professional accountability, AI audit, documentation standards, consent, procurement, and post-deployment monitoring. This is especially important in home-care and community nursing, where AI systems may connect patients, caregivers, nurses, general practitioners, hospitals, and service providers across organizational boundaries. Recent governance-focused healthcare AI literature emphasizes data readiness, model validation, workflow integration, institutional accountability, human oversight, post-deployment monitoring, and workforce readiness as core requirements for responsible AI integration [62,63,65,66,69,70]. Without robust governance and interoperable infrastructure, AI may increase fragmentation rather than improve care coordination.

### 4.4. Strengths

A key strength of this review is its broad and inclusive scope, capturing diverse AI applications across diagnosis, monitoring, decision support, and workflow enhancement in home-care and community nursing. By synthesizing evidence across these domains rather than focusing narrowly on a single technology, condition, or clinical task, the review provides a holistic overview of the current landscape and highlights the multiple ways AI may influence community-based nursing practice. Another strength is the review’s attention to both technological performance and implementation context. In addition to summarising reported benefits, the review integrates evidence on usability, interoperability, workload, regulatory concerns, and nursing practice implications. This approach provides a more practice-oriented synthesis that reflects not only what AI-enabled systems can do, but also the conditions under which they may be acceptable, feasible, and clinically meaningful in real-world community settings. The review therefore contributes by identifying emerging opportunities while also highlighting the practical, ethical, and professional considerations that should guide future AI development, evaluation, and implementation in community nursing.

### 4.5. Limitations

Several limitations of this review should be acknowledged when interpreting the findings. First, the search strategy may have limited the breadth of included evidence. Only English-language publications were included, which may have excluded relevant studies published in other languages and introduced language bias. Although the date restriction from 2020 onwards was intentionally applied to capture contemporary AI applications in a rapidly evolving field and is aligned with the review question, this decision may have excluded earlier studies relevant to the historical development of AI in home-care and community nursing. In addition, although major health and nursing databases were searched, relevant studies indexed in other databases, grey literature sources, conference proceedings, or unpublished implementation reports may have been missed. These language, date, database, and publication-source restrictions may have influenced the completeness of the evidence map and should be considered when interpreting the scope of available evidence. Second, this review did not include a formal quality appraisal of included studies, consistent with common scoping review methodology; however, this limits the ability to assess the methodological strength of individual studies or determine the extent to which study quality influenced the findings. Protocol registration was also completed retrospectively, which may reduce transparency regarding any changes made during the review process. Third, the evidence base was relatively small, heterogeneous, and context-specific. Included studies varied considerably in design, AI application, clinical focus, outcome measures, and community or home-care context, limiting direct comparison across studies and preventing firm conclusions about effectiveness or implementation readiness. Many studies were early-stage, used small samples, prototype systems, simulated data, or exploratory qualitative designs, increasing uncertainty and potential risk of bias. Therefore, the findings should be interpreted as an overview of emerging evidence and implementation considerations rather than definitive evidence of clinical effectiveness or sustained integration into community nursing practice.

## 5. Conclusions

This scoping review mapped emerging empirical evidence on AI applications in home-care and community nursing. The findings indicate that AI is being used or tested to support clinical assessment, remote monitoring, risk prediction, care coordination, and nursing workflow optimization. However, the evidence base remains limited, heterogeneous, and largely concentrated in development, validation, or early implementation studies. Accordingly, AI should be understood as having potential to support home-care and community nursing, while current evidence should be interpreted as emerging rather than definitive. Realizing this potential will require rigorous real-world evaluation, equity-focused validation, interoperable digital infrastructure, transparent governance, ethical safeguards, and meaningful engagement with nurses, patients, caregivers, and service organizations. Future research should examine not only technical performance, but also safety, acceptability, workflow impact, professional accountability, patient outcomes, equity, and sustainability. AI-enabled systems are most likely to strengthen community nursing when they augment nurses’ clinical judgement, preserve relational and person-centred care, and are embedded within accountable, human-centred models of care.

## Figures and Tables

**Table 1:** Characteristics of the included studies.

Author(s) Year of Publication	Country	Study Design	Study Aims	Setting	Sample Characteristics		Type of AI Technology Used (Purpose, Function, Stage of Implementation)	Key Findings	Challenges/Limitations	Implications
					Sample Size	Nurses/Caregivers Roles				
Karnehed S et al. (2025) [23]	Sweden	Exploratory qualitative study using semi-structured interviews (Gioia methodology)	To explore nurses’ perceptions of wound care in municipal home healthcare and the opportunities and challenges with the integration of AI technologies into their practices.	Municipal home healthcare (wound care context)	Patients requiring treatment for hard-to-heal wounds (e.g., pressure ulcers, diabetic foot ulcers).	Nurses: 14 Registered nurses working in municipal home healthcare with wound care experience.	AI technologies are discussed conceptually, specifically mentioning AI-based image recognition for wound assessment, automated medication dispensers, and digital decision support systems. Purpose: To explore nurses’ perceptions of opportunities and challenges posed by AI integration into wound care practices. Function: Potential use for documentation, wound image analysis, and enhancing decision-making. Stage: Early stages/Perception study (AI technologies mostly in pilot or development phases in Sweden).	Three interconnected dimensions emerged from the data: relational, embodied, and adaptive practices. Nurses emphasized the trust and continuity necessary for effective wound care, which AI-driven automation might overlook. Embodied practices were central to wound care, raising nurses’ concerns about AI’s ability to replicate these nuanced judgments. Adaptive practices were presented as challenges for AI integration, as	Existing digital systems were perceived as rigid and often increased administrative burdens rather than streamlining care.	Successful AI integration should account for the realities of nursing practice, ensuring that technological tools enhance the embodied, relational, and adaptive dimensions of wound care
Du Q et al. (2022) [31]	China	Quasi-experimental study	To evaluate the effect of functional magnetic resonance imaging (fMRI) under the fuzzy C-means (FCM) clustering algorithm on plan-do-check-action (PDCA) home nursing for patients with diabetic nephropathy (DN)	Home nursing (after discharge) and follow-up in the community setting	Intervention with PDCA home nursing (n = 32) Control group with routine home nursing (n = 32)	Nurses: Specially trained full-time nurses provided PDCA home nursing and follow-ups.	Improved Fuzzy C-means (FCM) clustering algorithm applied to Functional Magnetic Resonance Imaging (fMRI) data. Purpose: To evaluate the effect of PDCA home nursing; the improved FCM algorithm clustered activation regions to assist in diagnosis and reduce errors. Function: Image clustering and processing of fMRI images to detect activation points. Stage: Algorithm improvement, Application, and Evaluation.	The improved FCM algorithm detected the activation regions in the fMRI images more effectively.	Not reported	The FCM algorithm could provide assistance in diagnosis and reduce errors and misdiagnosis
Zhao H et al. (2025) [32]	China	Randomized controlled trial	To explore postoperative self-care ability of continuous nursing based on artificial intelligence for stroke patients with neurological injury	Continuous nursing from hospital to home, utilizing the WeChat platform and home care	Intervention group with AI-based care (n = 53); Control group with conventional (n = 53)	Nurses: Continuous nursing coaches composed of nurses and community medical staff. Patients: Stroke patients with neurological injury (postoperative/post-discharge).	Artificial Intelligence (AI) continuous nursing model combined with the WeChat platform. Purpose: To optimize continuing care, provide personalized guidance based on vital signs data, and improve postoperative self-care ability and quality of life. Function: Remote monitoring and personalized guidance/recommendations. Stage: Application/Intervention and Verification.	Compared with the control group, the patients’ self-care ability, depression state, compliance with health guidance and laboratory indicators were also better than those in the control group, and the differences were statistically significant (*p* < 0.05).	Not reported	Not reported
Kwon S-H et al. (2025) [33]	South Korea	Descriptive cross-sectional survey study (UTAUT framework)	To understand the behavioral intention to use digital healthcare and its influencing factors based on the Unified Theory of Acceptance and Use of Technology (UTAUT)	Home care (Hospital-based home care, Home Hospice, Community Health Center-based home care)	Patients managed for cancer, diabetes mellitus, cardiovascular, neurologic, respiratory, kidney, and liver diseases.	Nurses: 180 home care nurses providing home care services (HHC, HH, CHC).	Survey assessed behavioral intention to use Big data/AI solutions (e.g., overdose prevention), AI-enabled care sensors (e.g., fall detector), and AI companion robots. Purpose: To understand nurses’ behavioral intention to adopt various digital health technologies in home care. Function: Survey items covered future use of AI for solutions and sensors. Stage: Adoption/Intention stage.	Home care nurses generally showed strong behavioral intentions to adopt digital healthcare. Big data/artificial intelligence (AI) solutions (87.2%) and AI-assisted care sensors (85.6%) had the highest behavioral intention to use.	Digital health enhances accessibility and convenience; it cannot replace in-person interactions essential for personal engagement and holistic patient assessment. Greater concern for data privacy.	Digital healthcare technology design should prioritize automation and operational efficiency without increasing workload
van Veen S et al. (2025) [34]	Netherlands	Cross-sectional study	To develop and test a prediction model for pain in community-dwelling frail older people in palliative care	Community-dwelling; receiving assistance from home-care nursing	157 patients with primary conditions, such as cardiovascular diseases, cancer, nervous-/sensory system diseases.	Nurses: Community-care nurses assisted in data collection. Patients: Frail older people (aged 65+) in palliative care (life expectancy < 1 year).	Prediction model using Multivariable Logistic Regression. Purpose: To develop a prediction model for the presence of pain in community-dwelling frail older people based on symptoms (e.g., insomnia, fatigue). Function: Statistical model development/risk prediction. Stage: Model development and testing.	Model performance was indicated as fair, with a sensitivity of 0.74 and a positive predictive value of 0.80. Insomnia and fatigue are predictive symptoms for pain, especially in women and younger patients.	Not reported	Future research should focus on the external validation of this model to support in the early identification of pain in older palliative care patients and enhance its predictive accuracy.
Xia Q et al. (2025) [35]	China	Cross-sectional study	To develop a model using a machine learning approach that can effectively identify the quality of home care in communities	Community Health Service Centres (CHSCs) providing home care.	170 Community Health Service Centres. Patients: Home patients (quality evaluated based on outcomes like pressure ulcers and satisfaction).	Nurses: Professional nurses performing home care; qualification and allocation are key factors assessed.	Convolutional Neural Network (CNN) model. Purpose: To develop an intelligent evaluation model for rapidly and accurately grading the quality of home care (Grades A, B, C, D). Function: Classification model based on seven crucial variables (e.g., nurse qualification, emergency plans). Stage: Model development and application/evaluation.	The convolutional neural network model was built upon seven variables, which encompassed the qualification of home nursing staff, developing and practicing emergency plan to cope with different emergency rescues in home environment, being equipped with medication and supplies for first aid according to specific situations, assessing nutrition condition of home patients, allocation of the number of home nursing staff, cases of new pressure ulcers and patient satisfaction rate.	Although the model developed in this study can identify quality grades, it cannot intelligently match measures.	Promoting home care quality using a CNN model is valuable for economically underdeveloped areas and worth promoting in the nursing field.
Henderson Betkus et al., (2026) [36]	Canada	Cross-sectional survey	To understand community health nurses’ awareness, knowledge, and perceptions of AI in practice and gain insights to better involve them in AI	Community	NA	228 Community health nurses’ familiarity with AI and their perceptions about its effect	Emergence of AI applications in nursing, main sources of knowledge for learning AI, relationship between community nurses’ knowledge level of AI technologies and their perceptions of the effects of AI on clinical practice, professional accountability, and the usefulness of AI applications, AI competencies that are needed in community nursing practice were surveyed. Purpose: To understand community health nurses’ awareness, knowledge, and perceptions of AI in practice and gain insights to better involve them in AI. Function: Nursing involvement provides a relevant perspective and knowledge that influences their informed decisions, which ensures clinical relevance and accuracy of AI and related machine learning. Stage: Early stages/Perception study	Most respondents (89.9%) felt they welcomed technology into their practice. 39.6% of respondents felt uncomfortable with the development of AI.	Many respondents had concerns related To professional accountability if they accepted a wrong AI recommendation or if they dismissed a correct AI Recommendation.	Educational resources to ensure that community health nurses have a sound basis for AI in their practice, which would promote their comfort with AI.
Zolnoori et al., (2026) [37]	United States	Cross-sectional study	To develop and evaluate a speech-based screening algorithm for detecting mild cognitive impairment (MCI) using routine follow-up calls.	Home health care	114 participants aged over 60 years	Nurses: home health care patient–nurse communication was captured using the Saramonic Blink 658 device.	A speech-based screening algorithm. Purpose: To address the moderate performance of existing electronic health record algorithms. Function: To detect mild cognitive impairment in home health care patients. Stage: Application/Intervention and Verification.	The multimodal model combining follow-up call speech and OASIS variables achieved the best performance (AUC = 91.03), outperforming models based on CDR interviews (AUC = 80.67), follow-up calls alone (AUC = 81.09), and OASIS alone (AUC = 78.53).	Although cross-sectional findings are promising, larger longitudinal studies are needed to determine whether the observed speech features predict progressive cognitive decline rather than transient fluctuations.	Speech collected during brief, semi-structured follow-up phone calls with structured EHR data can effectively support early detection of MCI in home health care settings.
Uzun Y et al. (2025) [38]	Türkiye	Feasibility study (prototype development and testing in a simulated environment)	To explore the feasibility of automating the monitoring of respiratory exercises using an AI-supported incentive spirometry system designed	Designed for use in both hospital and home environments. Focuses on home-based nursing applications.	250 scenario-based patients (simulated data). Patients needing respiratory exercises (incentive spirometry) to reduce postoperative complications.	Nurses: System designed to reduce manual nursing workload and augment nurse oversight.	AI-supported incentive spirometry system using Machine Learning (ML) models (Random Forest, XGBoost, SVM, etc.) and Image Processing (tablet camera). Purpose: To automate the monitoring of respiratory exercises, track spirometer volume in real-time, and classify respiratory performance. Function: Real-time analysis, classification, and feedback. Stage: Prototype development and technical feasibility demonstration (Pilot study).	The ensemble methods demonstrated exceptional performance, achieving 100% accuracy and R2 = 1.0, with cross-validation mean accuracies exceeding 99.4%.	The performance of existing image processing algorithms can be sensitive to suboptimal real-world conditions. Data security and privacy concerns.	The utilization of AI-supported incentive spirometry system represents a significant step at the intersection of innovation and artificial intelligence in nursing care
Georgescu AL et al. (2024) [39]	Brazil	Observational study	To illustrate the preliminary transferability of two established AI models designed to detect high depression and anxiety symptom scores	Remote mental health screening; recruited via a company specializing in nurse-led, holistic home care (Laços Saúde)	69 Brazilian adults (aged 51–92 years) screened for depression (PHQ-8) and anxiety (GAD-7) symptoms.	Study supported by a nurse-led home care company.	Two Random Forest (RF) models. Purpose: To assess the preliminary transferability and generalizability of AI models used to screen for depression and anxiety symptoms remotely. Function: Binary classification based on performance features derived from a nonverbal working memory game (n-back task). Stage: Validation/Testing (testing a pre-trained model on a new demographic).	The depression classification model exhibited robust performance, achieving an area under the receiver operating characteristic curve (AUC) of 0.78, a specificity of 0.69, and a sensitivity of 0.72. The anxiety classification model showed an initial AUC of 0.63, with a specificity of 0.58 and a sensitivity of 0.64.	Results of the AI models are based on the cohort level; the ability to detect intraindividual changes in symptom severity across time is unknown.	AI tools may become powerful aids in remote screening and Monitoring, potentially contributing to a significant reduction in mental health underdiagnoses globally.
Rochon M et al. (2024) [40]	United Kingdom	Development and evaluation study (WISDOM AI study)	Using AI to prioritise surgical wound images for clinician review, aiming to efficiently manage workload.	Remote monitoring of surgical wounds for patients experiencing complications at home	Training dataset: 37,974 images; Testing dataset: 3634 images.	Nurses: Clinicians review patient-submitted images. Patients: Surgical patients experiencing post-operative complications (wound assessment/surgical site infection surveillance).	Artificial intelligence (AI) algorithm (WISDOM AI) using AI-driven image recognition. Purpose: To automatically analyze patient-submitted wound images to spot signs of conditions (e.g., infections) and prioritize urgent cases for clinician review, thereby reducing workload. Function: Image recognition, detection of dehiscence/inflammation, prioritization. Stage: Development and Evaluation.	The AI demonstrated a sensitivity of 89%, exceeding the target of 85% and proving effective in identifying cases requiring priority review. Intra-rater reliability was perfect, achieving 100% consistency in repeated assessments.	Challenges in identifying issues such as redness and discoloration in darker skin tones.	AI algorithm remote wound monitoring and improving healthcare outcomes for all patients.
Chae S et al. (2022) [41]	United States	Retrospective study	To identify home health care (HHC) patients with heart failure who have poor self-management, and to examine patient factors associated with poor self-management.	HHC agency	9710 Heart Failure (HF) patients	HHC clinicians (registered nurses, social workers, therapists, managers) provided narrative notes.	Natural Language Processing (NLP) algorithm. Purpose: To extract documentation of poor self-management from free-text HHC narrative notes. Function: Analyzes text data to identify six domains of poor self-management (e.g., poor diet adherence, missed appointments). Stage: Application and Validation.	1. Patients with HF who have poor self-management can be identified from narrative notes using NLP. 2. Male gender, diagnosis of diabetes or depression, impaired decision-making, smoking, and shortness of breath with exertion are associated with poor self-management.	NLP algorithm may miss some information about patient self-management or may misclassify notes.	NLP techniques in this study can be used to develop computerized decision support systems to make use of this rich data to aid clinicians in identifying patients with documented poor self-management. This might save time and reduce clinical documentation review burden.
Lee J et al. (2023) [42]	South Korea	System development study involving focus group interviews and a satisfaction survey	To develop a web application that reflects the needs of visiting nurses.	Visiting health services/Home-visiting services in the community (CARE-Net system)	Patients: Community-dwelling frail older adults; chronic disease management (hypertension/diabetes) and frailty management were target uses.	Nurses: Visiting nurses of public health centers and community centers (6 nurses + 21 nurses/managers).	Artificial Intelligence (AI) algorithm incorporated into the CARE-Net system. Purpose: To develop a feature that recommends health and welfare services (linked service recommendation system) tailored to each patient’s health status. Function: AI-based linked service recommendation system. Stage: Development and Evaluation.	CARE-Net system can be used with mobile devices to increase the mobility of visiting nurses	Not reported	CARE-Net could help in the effective case management of community-dwelling older adults. This would also reduce nurses’ workload and enhance effective referrals between healthcare and long-term care institutions
Cheng J et al. (2025) [43]	China	Two-wave survey utilizing Structural Equation Modeling (SEM) and Artificial Neural Network (ANN) analysis	To investigate what and how the factors affect nurses’ use of Mobile Home Nursing Care Applications (MHNCAs)	Home nursing care services; connecting nurses with patients online via MHNCAs	Elderly patients with chronic noncommunicable diseases (implied).	First wave: 836 nurses with 807 valid answers Second wave: 512 nurses with 445 valid answers	Artificial Neural Network (ANN) analysis. Purpose: To validate a model based on social cognitive theory and determine the relative importance of factors affecting nurses’ use of MHNCAs. Function: Predictive modelling of nurses’ behavioral intention to use MHNCAs. Stage: Modeling and Evaluation.	1. Factors and their working mechanisms of nurses’ use of MHNCAs are uncovered and validated through the SEM and ANN. 2. Environmental factors could affect nurses’ use of MHNCAs.	Not reported	1. The proposed model could be validated in other countries. 2. Results of model applied in this study can benefit nursing managers, nursing educators, and the broader nursing community by informing the design of MHNCAs tailored for nurses.
Lim et al. (2026) [44]	South Korea	Retrospective cohort study	To identify high-risk factors for type 2 diabetes and develop a machine learning (ML)–based diabetes prediction model using nursing notes from home health care.	Home health care	7896 medical records from 1747 patients aged ≥ 20 years who received home health care over 10 years	Nurses: community health nurses playing a critical role in identifying early health risks during home visits, provided nursing notes	Natural Language Processing (NLP) algorithm. Purpose: To extract diabetes risk factors from home health nursing records. Function: Female sex, depression, hypertension, hyperlipidemia, nursing services such as dressing and nutritional care, and dysuria were identified as prediabetes-related symptoms extracted from nursing notes. Stage: Application and Validation	Female sex, depression, hypertension, hyperlipidemia, nursing services such as dressing and nutritional care, and dysuria were identified as prediabetes-related symptoms extracted from nursing notes. The Random Forest model demonstrated the highest predictive performance (AUC = 0.985).	While the SMOTE filter improves the algorithm’s performance, it is important to consider the potential risk of overfitting.	Future research should validate this model across diverse home healthcare settings to enhance its generalizability and practical applicability.
Firima E et al. (2024) [45]	Lesotho	Cross-sectional study	To examine the proportion of evaluable echocardiograms obtained using a pragmatic approach involving image acquisition by short-term trained nurses and nurse assistants with hand-held ultrasound devices, followed by AI-supported interpretation.	Community-based care; focused echocardiography performed at participants’ homes in hard-to-reach areas	756 individuals with elevated blood pressure, assessed for Left Ventricular Hypertrophy (LVH).	16 nurses/nurse-assistants with short-term training.	Deep learning algorithms (AI-supported analysis). Purpose: To provide automated interpretation of focused echocardiograms (PLAX views) obtained by non-physician staff to improve LVH diagnosis/monitoring in resource-limited settings. Function: AI-powered interpretation of cardiac images captured by a hand-held ultrasound device. Stage: Implementation and Evaluation.	It is feasible that non-physician healthcare workers can evaluate and monitor LVH in resource-limited settings.	AI algorithm could not evaluate some echocardiograms.	An approach involving short-term trained nurses and nurse assistants, followed by AI-supported interpretation could solve an unmet clinical need in hard-to-reach Environments.

## Data Availability

No new data were created or analyzed in this study. Data sharing is not applicable to this article.

## Supplementary Materials

The following supporting information can be downloaded at: https://www.mdpi.com/article/10.3390/healthcare14183034/s1, Supplementary File S1: Database-specific search strategies and corresponding search yields for PubMed, CINAHL via EBSCO, Embase, Web of Science, PsycINFO via EBSCOhost, Scopus, Ovid, and ProQuest.

## Abbreviations

The following abbreviations are used in this manuscript:

AI	Artificial Intelligence
JBI	Joanna Briggs Institute
LVH	Left Ventricular Hypertrophy
IoT	Internet of Things
CBPHC	Community-Based Primary Health Care
CFIR	Consolidated Framework for Implementation Research
RE-AIM	Reach, Effectiveness, Adoption, Implementation, Maintenance
