# Supplementary material for: Artificial Intelligence (AI) in Home-Care and Community Nursing: A Scoping Review
Source: Healthcare (Basel). 2026 Sep 16;14(18):3034. doi: 10.3390/healthcare14183034 (PMC13606495; doi:10.3390/healthcare14183034)
Supplement: Supplementary file 1 [file healthcare-14-03034-s001.zip › healthcare-4459953-supplementary.pdf]

Searching results 14/05/2026

PubMed

#1	((Nurses[MeSH Terms]) OR (Nurse*[Title/Abstract]))	416,388
#2	((Artificial Intelligence[MeSH Terms]) OR (Deep Learning[MeSH Terms])) OR (Machine Learning[MeSH Terms])	281,559
#3	(((((((((((((((((Intelligence, Artificial[Title/Abstract])) OR (Machine Learning[Title/Abstract])) OR (Artificial Intelligence[Title/Abstract])) OR (artificial intelligen*[Title/Abstract])) OR (AI[Title/Abstract])) OR (big learning[Title/Abstract])) OR (deep learning[Title/Abstract])) OR (automation[Title/Abstract])) OR (cognitive computing[Title/Abstract])) OR (neural networks[Title/Abstract])) OR (intellig* computing[Title/Abstract])) OR (natural language processing[Title/Abstract])) OR (chatbot*[Title/Abstract])) OR (chatter robot[Title/Abstract])) OR (conversational agent[Title/Abstract])) OR (expert system[Title/Abstract])) OR (Robotics[Title/Abstract])	495,213
#4	#2 OR #3	595,997
#5	(Home Care Services[MeSH Terms]) OR (Community Health Nursing[MeSH Terms])	69,764
#6	(((((home care[Title/Abstract]) OR (Home Health Nursing[Title/Abstract])) OR (home healthcare[Title/Abstract])) OR (district nurs*[Title/Abstract])) OR (community nurs*[Title/Abstract])) OR (community car*[Title/Abstract])	40,160
#7	#5 OR #6	92,023
#8	#1 AND #4 AND #7	73
#9	#1 AND #4 AND #7 Filters: from 2000 - 2026	70

CINAHL via EBSCO

#1	SU (Nurses) OR AB (Nurse*)	692,489
#2	SU (artificial intelligence) OR SU (Deep Learning) OR SU (Machine Learning)	284,809
#3	MM (artificial intelligence or ai or a.i. or machine learning or deep learning) OR AB (conversational agent) OR AB (machine learning) OR AB (artificial intelligen*) OR AB (big learning) OR AB (deep learning) OR AB (automation or robot or ai or artificial intelligence) OR AB (cognitive computing) OR AB (neural networks) OR AB (intellig* computing) OR AB (natural language processing) OR AB (chatbot or virtual agent or virtual assistant or bot)	616,605
#4	S#2 OR #3	680,454
#5	SU (Home Care Services) OR SU (Community Health Nursing) OR AB (home care) OR AB (Home Health Nursing) OR AB (home healthcare) OR AB (district nurs*) OR AB (community nurs*) OR AB (community car*)	216,801
#6	#1 AND #4 AND #5	165
#7	#1 AND #4 AND #5 01/01/2020 - 05/31/2026	117

Embase

**Search Items**	**Search strategy**	
#1	**'nurse'**/exp OR **'nurse'** OR **nurse***:ab,ti	644,003
#2	**'artificial intelligence'**/exp OR **'artificial intelligence'** OR **'machine learning'**:ti,ab,kw OR **'deep learning'**:ti,ab,kw OR **ai**:ti,ab,kw OR **'intelligence, artificial'**:ti,ab,kw OR **'big learning'**:ti,ab,kw OR **automation**:ti,ab,kw OR **'cognitive computing'**:ti,ab,kw OR **'neural networks'**:ti,ab,kw OR **'intellig* computing'**:ti,ab,kw OR **chatbot***:ti,ab,kw OR **'natural language processing'**:ti,ab,kw OR **'chatter robot'**:ti,ab,kw OR **'conversational agent'**:ti,ab,kw OR **'expert system'**:ti,ab,kw OR **robotics**:ti,ab,kw	703,955
#3	**'home care'**/exp OR **'home care'** OR **'home care services'**:ti,ab,kw OR **'community health nursing'**:ti,ab,kw OR **'home health nursing'**:ti,ab,kw OR **'district nursing'**:ti,ab,kw OR **'home healthcare'**:ti,ab,kw OR **'community nursing'**:ti,ab,kw OR **'cognitive computing'**:ti,ab,kw OR **'community car*'**:ti,ab,kw	132,382
#4	#1 AND #2 AND #3	146
#5	#4 AND (**2020**:py OR **2021**:py OR **2022**:py OR **2023**:py OR **2024**:py OR **2025**:py OR **2026**:py)	124

Web of science

Search Items	Search strategy	
#1	(AB=(Nurses)) OR AB=(Nurse*)	396,077
#2	((TS=(Deep Learning)) OR TS=(Artificial Intelligence)) OR TS=(Machine Learning)	1,394,623
#3	((((((((((((((AB=(Intelligence, Artificial)) OR AB=(Machine Learning)) OR AB=(Artificial Intelligence)) OR AB=(artificial intelligen*)) OR AB=(AI)) OR AB=(big learning)) OR AB=(deep learning)) OR AB=(automation)) OR AB=(cognitive computing)) OR AB=(neural networks)) OR AB=(intellig* computing)) OR AB=(natural language processing)) OR AB=(chatbot*)) OR AB=(chatter robot)) OR AB=(conversational agent)) OR AB=(expert system)) OR AB=(Robotics)	2,263,600
#4	#2 OR #3	2,472,405
#5	(((((((TS=(Home Care Services)) OR TS=(Community Health Nursing)) OR AB=(home care)) OR AB=(Home Health Nursing)) OR AB=(home healthcare)) OR AB=(district nurs*)) OR AB=(community nurs*)) OR AB=(community car*)	576,051
#6	#1 AND #4 AND #5	1,686
#7	#1 AND #4 AND #5 and 2026 or 2025 or 2024 or 2023 or 2022 or 2021 or 2020 (Publication Years)	1229

PsycINFO via EBSCOhost

Search Items	Search strategy	
#1	MA (Nurses) OR XB (Nurse*)	91,793
#2	MA (artificial intelligence or ai or a.i. or machine learning or deep learning) OR XB (conversational agent) OR XB (machine learning) OR XB (artificial intelligen*) OR XB (big learning) OR XB (deep learning) OR XB (cognitive computing) OR XB (neural networks) OR XB (natural language processing) OR XB (intellig* computing) OR XB (chatbot) OR XB (virtual agent)	58,905
#3	MA (home care) OR XB (Home Care Services) OR XB (Community Health Nursing) OR XB (home care) OR XB (Home Health Nursing) OR XB (home healthcare) OR XB (district nurs*) OR XB (community nurs*) OR XB (community car*)	53,363
#4	#1 AND #2 AND #3	17
#5	#1 AND #2 AND #3 2020/01/01 - 2026/05/31	12

Scopus

Search Items	Search strategy	
#1	(TITLE-ABS-KEY (nurse) OR TITLE-ABS-KEY (nurses))	556,289
#2	(TITLE-ABS-KEY (Artificial Intelligence) OR TITLE-ABS-KEY (Machine Learning) OR TITLE-ABS-KEY (Deep Learning) OR TITLE-ABS-KEY (Machine Learning) OR TITLE-ABS-KEY (cognitive computing) OR TITLE-ABS-KEY (intellig* computing) OR TITLE-ABS-KEY (natural LANGUAGE processing) OR TITLE-ABS-KEY (conversational agent) OR TITLE-ABS-KEY (chatter robot) OR TITLE-ABS-KEY (chatbots))	2,607,668
#3	(TITLE-ABS-KEY (home care) OR TITLE-ABS-KEY (Home Care Services) OR TITLE-ABS-KEY (Community Health Nursing) OR TITLE-ABS-KEY (Machine Learning) OR TITLE-ABS-KEY (Home Health Nursing) OR TITLE-ABS-KEY (home healthcare) OR TITLE-ABS-KEY (district nursing) OR TITLE-ABS-KEY (community nursing) OR TITLE-ABS-KEY (community car*))	2,315,980
#4	#1 AND #2 AND #3	1599
#5	#1 AND #2 AND #3 AND (LIMIT-TO (PUBYEAR , "2020") OR LIMIT-TO (PUBYEAR , "2021") OR LIMIT-TO (PUBYEAR , "2022") OR LIMIT-TO (PUBYEAR , "2023") OR LIMIT-TO (PUBYEAR , "2024") OR LIMIT-TO (PUBYEAR , "2025") OR LIMIT-TO (PUBYEAR , "2026"))	1310

Ovid

#1	(Nurses or Nurse*).mp. [mp=title, abstract, full text, caption text]	25139
#2	("Home Care Services" or "Community Health Nursing" or "home care" or "Home Health Nursing" or "home healthcare" or "district nursing" or "community nursing" or "community caring").mp. [mp=title, abstract, full text, caption text]	4309
#3		34

	("Artificial Intelligence" or "Intelligence, Artificial" or "Machine Learning" or "artificial intelligen*" or "AI" or "big learning" or "deep learning" or "automation" or "cognitive computing" or "neural networks" or "intellig* computing" or "natural language processing" or "chatbot*" or "chatter robot" or "conversational agent" or "expert system" or "Robotics").m_titl.	
#4	("Artificial Intelligence" or "Intelligence, Artificial" or "Machine Learning" or "artificial intelligen*" or "AI" or "big learning" or "deep learning" or "automation" or "cognitive computing" or "neural networks" or "intellig* computing" or "natural language processing" or "chatbot*" or "chatter robot" or "conversational agent" or "expert system" or "Robotics").mp. [mp=title, abstract, full text, caption text]	227
#5	#3 OR #4	227
#6	#1 AND #2 AND #5	40
#7	limit 6 to yr="2020 -Current"	19

ProQuest

#1	subject(nurses') OR summary(nurse)	2,160,366
#2	subject('artificial AND intelligence') OR summary(Machine Learning) OR summary(artificial intelligen*) OR summary(big learning) OR summary(deep learning) OR summary(AI) OR summary(chatbot*) OR summary(natural language processing) OR summary(conversational agent) OR summary(cognitive computing)	4,254,390
#3	summary(home care) OR summary('community and health and nursing') OR summary(Home Health Nursing) OR summary(community of caring) OR summary(community nursing) OR summary(home healthcare) OR summary(Home Care Services) OR summary(Community Health Nursing)	1,284,628
#4	#1 AND #1 AND #3	620
#5	#1 AND #1 AND #3Publication date 2020-01-01 - 2026-05-14	373
